# A G-Code-Driven Modeling and Thermo-Mechanical Coupling Analysis Method for the FDM Process of Complex Lightweight Structures

**DOI:** 10.3390/ma19061200

**Published:** 2026-03-18

**Authors:** Dinghe Li, Yiheng Dun, Zhuoran Yang, Rui Zhou, Yuxia Chen

**Affiliations:** 1 Sino-European Institute of Aviation Engineering, Civil Aviation University of China, Tianjin 300300, China; zzc7465221@163.com (Y.D.); jmyang@cauc.edu.cn (Z.Y.); 19903131815@163.com (Y.C.); 2 Aeronautical Engineering Institute, Civil Aviation University of China, Tianjin 300300, China; 18722361207@163.com

**Keywords:** FDM, G-code-driven modeling, transient thermal analysis, thermo-mechanical coupling, element birth–death method, residual stress, warpage deformation

## Abstract

Accurate prediction of thermo-mechanical behavior in Fused Deposition Modeling (FDM) is often limited by mismatches between idealized Computer-Aided Design (CAD) geometry and path-dependent material deposition. This paper presents a G-code-driven, filament-level modeling and process-simulation workflow for complex geometries and infill strategies, especially toolpaths with in-plane inclinations. Extrusion segments are parsed from slicing G-code to obtain endpoints and process parameters, and each filament is reconstructed as a path-aligned rectangular bead using a dedicated local coordinate system. Progressive deposition is simulated in ANSYS Parametric Design Language (APDL) via an element birth–death method, enhanced by a centroid-based element selection strategy that reduces dependence on strictly aligned hexahedral partitions and improves robustness for complex meshes. A nonlinear transient thermal analysis is performed, and temperatures are mapped to the structural model through an indirect thermo-mechanical coupling scheme to predict warpage and residual stresses. Case studies on square plates with triangular and hexagonal infills (with/without sidewalls and a bottom base) show that the high-temperature zone follows newly deposited paths with peak temperatures near 220 °C, while displacement and von Mises stress accumulate and are strongly affected by infill topology and boundary conditions.

## 1. Introduction

Against the backdrop of mass customization in the era of Industry 4.0, the demand for structures integrating lightweight design and high performance has grown increasingly prominent. By means of lattice design, topology optimization and other approaches, it is feasible to decrease component weight and material consumption while preserving their mechanical properties. However, conventional subtractive manufacturing methods and injection molding processes are confronted with limitations when it comes to fabricating components with complex internal structures or functionally graded characteristics. With the continuous advancement of Additive Manufacturing (AM) technologies, a host of complex configurations that were previously unachievable through traditional manufacturing methods have been afforded new forming routes.

FDM ranks among the most extensively adopted polymer AM technologies. Its fundamental principle lies in heating thermoplastic filaments to a molten state, depositing the material layer by layer along the predefined paths, and ultimately fabricating a three-dimensional structure. Owing to its layer-by-layer deposition and path-controlled characteristics, FDM parts generally exhibit prominent issues such as anisotropy, residual stress concentration, and warpage deformation. These defects have to a certain extent restricted its application in load-bearing structures. Therefore, conducting high-fidelity numerical simulations to reveal the evolution laws of temperature fields and stress fields during the FDM process has become a crucial research direction in this field.

Recent experimental studies have systematically quantified how process parameters govern mechanical responses across both solid coupons and architected structures. For example, Tang et al. [1] combined full-field strain measurement (e.g., digital image correlation) and microstructural observations to reveal parameter-dependent trade-offs between the tensile behavior of Polymerized Lactic Acid (PLA) specimens and the compressive response of PLA lattice structures, highlighting distinct strain-field evolution and failure modes in lattices. Ben Hadj Hassine et al. [2] further demonstrated that infill pattern, infill density, and cell orientation can markedly alter tensile performance for ASTM-standard PLA specimens, supporting the need for structured parameter screening rather than ad hoc tuning. Complementarily, Sahoo et al. [3] linked layer thickness, raster angle, and build orientation to Acrylonitrile Butadiene Styrene (ABS) mesostructure (void content and bead fusion) and tensile strength using Design of Experiments (DOE)/Analysis of Variance (ANOVA), establishing a clear process-mesostructure-property pathway for strength improvement.

Optimization-oriented works have advanced from single-objective tuning to trade-off-aware decision making. Zaman et al. [4] and Weakea et al. [5] applied Taguchi-based DOE to identify dominant parameters for strength enhancement and to provide statistically grounded parameter selection workflows. Ramadan et al. [6] proposed a multi-objective framework that couples DOE (Taguchi/response surfaces) with mixed-integer nonlinear optimization to simultaneously maximize tensile strength while minimizing energy consumption, demonstrating the practicality of Pareto-style tuning for FDM process planning. In addition, Aldosari et al. [7] used finite element simulations to compare fully solid and partially filled (e.g., 60% infill) PLA parts, reinforcing that appropriate infill design can reduce material usage while maintaining near-solid mechanical performance in selected loading scenarios.

Interlayer bonding remains a central bottleneck for load-bearing FDM components due to incomplete polymer chain interdiffusion, interfacial voids, and thermal-history-induced anisotropy. Mechanism-driven modeling by Gurrala and Regalla [8] established a viscous-sintering-based neck-growth framework that links filament coalescence dynamics to evolving tensile load capacity, and experimental validation indicated that typical processing often yields partial rather than fully healed interfaces. At the interface scale, Perez et al. [9] designed specimens to isolate interlayer interface strength and combined experiments with cohesive-zone modeling to show that interfacial debonding and residual gaps can dominate brittle failure, suggesting that reducing interlayer voids is a direct route to stiffness/strength gains.

On the enhancement side, Omer et al. [10] synthesized interlayer-bonding improvement technologies into pre-printing, in-printing, and post-printing categories (including feedstock modification, in situ interventions such as preheating or vibration/ultrasonics, and post-processing such as annealing/welding), while emphasizing that hybrid strategies are likely required to achieve robust structural reliability. Specific material-structure tailoring has also proven effective: Shen et al. [11] improved ABS interlayer bonding by engineering polymer molecular/phase structures to increase melt fluidity and chain diffusion/entanglement, and directly quantified improvements via peel-type testing. Environmental control is another strong lever; Kumrai-Woodruff and Wang [12] showed that elevating and stabilizing ambient/chamber temperature can significantly enhance Z-direction strength in ABS by reducing heat loss and promoting interlayer diffusion, which was corroborated through thermal imaging and microscopy-based bond-quality assessment.

High-fidelity simulation has increasingly been used to interpret process-induced thermal gradients, residual stresses, and macroscopic distortion, with particular emphasis on computational strategies that can represent progressive deposition. Houmimi et al. [13] integrated multiphysics simulation with thermal-camera measurements to quantify how printing speed and layer thickness affect residual stress levels and surface texture/morphology in PLA, illustrating the sensitivity of thermal history to process settings. A series of thermo-structural coupled models based on EBDM has been developed to simulate the layerwise addition of material: Chen et al. [14] and Yang and Zhang [15] reported coupled temperature-stress frameworks capable of predicting warpage trends and comparing path/filling strategies; Yang et al. [16] proposed a path-based discrete modeling method to predict warpage for complex geometries; and Yu et al. [17] treated infill line directions as a critical parameter and showed trade-offs between warpage severity and tensile/bending performance, providing guidance for distortion-strength balancing via infill-path design.

To improve efficiency and model construction robustness, Zhou et al. [18] proposed voxelization-based meshing and automatic sequencing methods for thermo-mechanical simulation and parameter optimization, while Garzon-Hernandez et al. [19] introduced a thermodynamically consistent constitutive framework for FDM thermoplastics that captures nonlinear, anisotropic, and rate-dependent behavior with explicit links to porosity and raster-induced anisotropy, enabling macroscopic structural prediction without explicitly resolving the entire deposition process. These efforts collectively indicate an ongoing shift from qualitative distortion prediction toward quantitatively reliable, path- and material-aware simulation frameworks.

A prominent trend in recent years is the move from idealized CAD-based models toward as-manufactured, toolpath-informed representations that better capture filament-scale geometry, raster anisotropy, and local defects. Hachimi et al. [20] developed workflows that convert FDM G-code into Abaqus scripts to reproduce printed geometries for mechanical simulation and reported high agreement between simulated and experimental tensile responses across raster orientations. Bacciaglia et al. [21] proposed a reproducible procedure to reconstruct 3D CAD geometry from G-code, enabling downstream Finite Element Analysis (FEA) on toolpath-informed structures while also documenting practical limitations and research needs. More broadly, Behseresht et al. [22] reviewed numerical modeling developments in fused filament fabrication, emphasizing persistent gaps in integrating melt flow, heat transfer/solidification, and structural analysis into unified predictive pipelines.

Toolpath-driven simulation concepts are not exclusive to polymer FDM. Mashhood et al. [23] demonstrated a G-code-driven thermo-mechanical Finite Element (FE) platform (in metal AM) that activates elements according to scan paths to predict thermal deformation and residual stresses, underscoring the general value of direct toolpath interfacing for process-aware prediction. Related path-conversion tools have also been explored in adjacent manufacturing simulations (e.g., incremental forming) by Nasulea and Oancea [24] as well as Gahbiche et al. [25], suggesting transferable methodologies for converting tool trajectories into analysis-ready boundary conditions or activation sequences.

In parallel with process and simulation advances, the material and functional scope of FDM continues to expand. Rahmatabadi et al. [26] applied response-surface methods to evaluate shape-memory behavior in PLA-TPU blends and found that infill density can dominate fixity and recovery metrics, supporting parameter-informed design for 4D-printing-oriented structures. Dul et al. [27] explored bicomponent core-sheath filaments (via co-extrusion/overcoating) to improve printability and mechanical performance, particularly under off-axis raster conditions where conventional monofilaments often underperform. At the composite/secondary-processing frontier, He et al. [28] investigated copper-tin-diamond composites produced via FDM-and-sintering, showing how reinforcement concentration and particle size drive porosity and microstructure and, in turn, mechanical performance and tool wear behavior.

Mechanical performance characterization has also broadened beyond quasi-static testing. Balasubramanian et al. [29] reported strain-rate-sensitive trade-offs among tensile, flexural, and interlaminar strengths in FDM-fabricated PLA, highlighting that testing protocols should reflect intended service loading. Ziemian and Ziemian [30] studied tension-tension fatigue of additively manufactured ABS across mesostructure layups and proposed a residual-strength degradation model, contributing to the reliability assessment of FDM parts under cyclic loading. Sheikh and Behdinan [31] combined experiments with a multi-scale model to predict properties of ABS and ABS-CNT systems and quantified how process parameters affect stiffness and bonding quality. Santos et al. [32] modeled filament temperature evolution and validated thermal histories via infrared thermography, providing data/benchmarks that support the calibration of more comprehensive thermal-mechanical models.

Macro-level manufacturing evolution is opening additional research directions. Tang et al. [33] reviewed multi-axis additive manufacturing, emphasizing that additional degrees of freedom can mitigate staircase effects and enable load-aligned toolpathing. Wirth et al. [34] derived scaling relationships among build rate, minimum feature size, and build volume across AM platforms, offering a broader perspective on performance limits and motivating toolpath- and process-aware strategies for future high-throughput yet high-fidelity manufacturing.

Most existing studies model and analyze FDM parts as an integral continuum or perform simplifications at the geometric level, making it difficult to fully reflect the influence of real printing paths and filament-level deposition behavior on thermo-mechanical responses. Meanwhile, there remains a lack of universal and efficient methods for directly mapping CAM models or G-codes generated by slicing software to FEA models. Based on this, this paper proposes a G-code-driven filament-level modeling method for complex geometries and complex filling strategies, and realizes the indirect thermo-mechanical coupling simulation of the FDM process via the ANSYS APDL 2022 R1. This work aims to provide a more refined numerical analysis tool for characterizing the temperature-field evolution during FDM processes and for predicting part warpage deformation and residual stresses.

The remainder of this paper is organized as follows. Section 2 outlines the overall research framework and the G-code-driven filament-level modeling workflow. Section 3 presents the theoretical formulation and numerical implementation, including the path-aligned local coordinate construction, filament geometry reconstruction, centroid-based element activation for element birth–death simulation, and the indirect thermo-mechanical coupling strategy. Section 4 provides numerical studies on representative lightweight plates with different infill topologies and boundary configurations, and discusses the resulting temperature evolution, warpage, and residual stress. Section 5 concludes the paper and highlights future research directions.

## 2. Methods

### 2.1. Research Framework

This study proposes a G-code-driven modeling and thermo-mechanical coupling analysis method for the FDM process, focusing on complex printing paths with inclined angles within the printing plane. Unlike the traditional FEA based on ideal CAD models and process simulation methods only applicable to parallel or perpendicular paths, the proposed method is directly driven by G-code generated in the slicing stage to construct an FE model that accurately matches the printing paths. Therefore, the mesh generation and activation of elements are more suitable for complex printing paths, enabling an accurate simulation of the evolution characteristics of transient temperature field, stress field, and displacement field.

**Figure 1:**
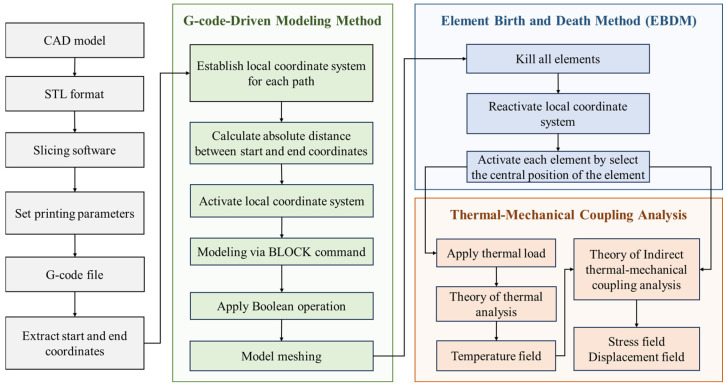
Framework of G-code-driven modeling and thermo-mechanical coupling analysis method for FDM process.

Figure 1 illustrates the framework of the proposed method, which consists of two parts: a G-code driven modeling method based on the local coordinate system and a thermo-mechanical coupling analysis method applicable to complex printing paths. First, a G-code file containing printing parameters and nozzle movement information is generated by slicing software. Subsequently, the G-code is processed to extract the printing paths where material extrusion actually occurs. For each path, a local coordinate system consistent with the printing direction is introduced to realize the automatic filament modeling. On this basis, the mesh generation of the current filament model is completed. Then, elements are gradually activated according to the path sequence. Meanwhile, transient thermal analysis and indirect thermo-mechanical coupling analysis are performed to obtain the evolution laws of the temperature field, stress field, and displacement field during the printing process. Each part of the method will be described in the following sections.

### 2.2. G-Code-Driven Modeling Method

FDM is a typical layer-by-layer material deposition process, whose printing process is directly controlled by the printing paths planned by slicing software. Printing paths not only determine the geometric morphology but also exert an influence on the material’s heating duration, cooling rate, as well as the final residual stress and warpage deformation. Therefore, printing paths serve as the key carrier connecting printing parameters, part geometries, and multi-physical field evolutions.

Based on the above understanding, this paper proposes a G-code-driven modeling method, which sufficiently utilizes printing paths extracted from G-code and constructs an FE model for the simulation of the printing process. This strategy avoids the problem of repeatedly constructing CAD models when printing parameters or path planning change, and is particularly suitable for studying thermo-mechanical coupling behaviors that are highly sensitive to paths. In addition, unlike the authors’ previous research work in [17] that mainly targets parallel or perpendicular paths, the proposed method considers the universal modeling requirements for printing paths with inclined angles, laying a methodological foundation for other mainstream AM processes.

#### 2.2.1. Establishment of the Local Coordinate System

**Figure 2:**
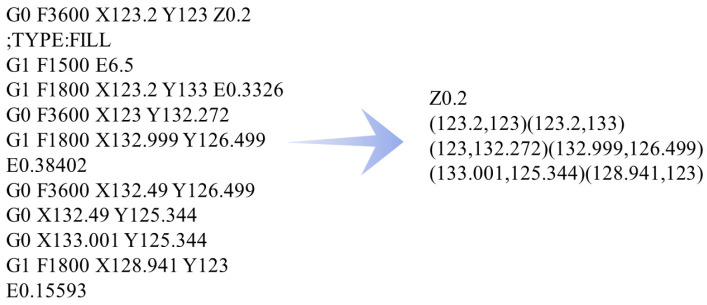
Extraction of filament-segment endpoints from raw G-code.

Referencing the work of Yang et al. in [16], it is necessary to process the G-code file generated by the slicing software. G-code incorporates a diverse set of instructions, including nozzle traversal, heating control, filament retraction, and material extrusion, among which only the extrusion-associated instructions are relevant to geometric modeling and process simulation. Accordingly, the original G-code is first scanned line by line. A C program developed by [16] is then employed to filter out the movement instructions corresponding to material extrusion, while extracting the start and end coordinates of nozzle movement. After that, the extracted coordinates are converted into a set of printing paths in chronological order. Figure 2 illustrates an example of converting the raw G-code text into a text file containing the start–end coordinate information of filament segments. Each path is characterized by its start point, end point, and corresponding process parameters (e.g., line width, layer height), which serves as the input for subsequent geometric modeling.

**Figure 3:**
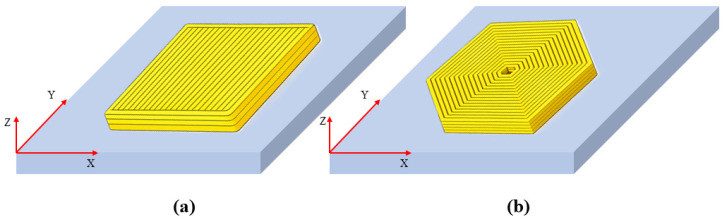
Different printing-path models: (**a**) Printing-path model aligned with the global coordinate system; (**b**) Printing-path model with paths distributed in arbitrary directions.

**Figure 4:**
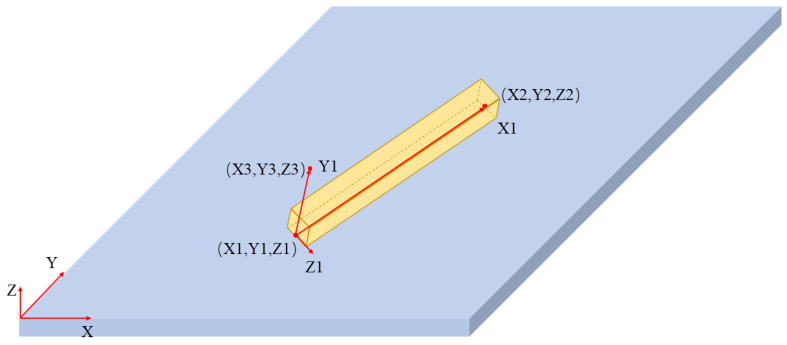
Schematic of establishing the local coordinate system for a single filament segment.

Note that the start and end coordinates of material extrusion are initially defined in the global coordinate system. For printing paths parallel to the axes of the global coordinate system, as shown in Figure 3a, geometric modeling can be directly implemented in this global coordinate system, and this part of the work has already been accomplished by Yang et al. [16]. However, when the paths are inclined or distributed in arbitrary directions, as shown in Figure 3b, modeling directly in the global coordinate system will significantly increase the complexity of geometric construction and numerical processing. To address this issue, this paper proposes a path-based local coordinate system modeling method by integrating the WPLANE command in APDL. Figure 4 illustrates the local coordinate system established on a single printed filament segment. Specifically, an independent local coordinate system is established for each printing path by executing the command “WPLANE, WN, X1, Y1, Z1, X2, Y2, Z2, X3, Y3, Z3”, where point A (X1, Y1, Z1) denotes the origin, the vector pointing from point A to point B (X2, Y2, Z2) defines the *X*-axis, and the vector pointing from point A to point C (X3, Y3, Z3) defines the *Y*-axis.

As mentioned earlier, the printing start and end coordinates of each filament in the global coordinate system have already been obtained. Herein, the start coordinate is designated as point A, with the X and Y coordinates corresponding directly. For the Z1 coordinate (i.e., the Z-coordinate of point A), its value of the local coordinate system for each layer is set to the current layer height minus a single layer height. It indicates that Z1 is uniformly set to 0 for the first layer. The end coordinate is designated as point B, with the X and Y coordinates also corresponding directly, and the Z2 value (i.e., the Z-coordinate of point B) is the same as Z1. Regarding point C, an assumption is first clarified that the extruded filament model is equivalent to a cuboid model, so as to ensure the continuity of heat conduction in subsequent thermo-mechanical coupling analysis. The cuboid width and height are set to the slicer line width and layer height, respectively, so that the deposited volume is consistent with the nominal material deposition defined by the printing parameters. While the real bead cross-section is typically flattened or approximately elliptical due to nozzle compression and interlayer contact, the cuboid approximation is adopted as a pragmatic representation of the toolpath-level deposition geometry. This simplification may affect the local surface-to-volume ratio and thus local convective heat loss, and the stress concentration near sharp corners at filament intersections. As a consequence, local peak temperature gradients or peak stresses at intersections may deviate from those obtained using a smoother (e.g., elliptical) cross-section. Nevertheless, the present work focuses on the comparative influence of deposition sequence and infill topology on the overall thermal history, warpage, and residual-stress distribution. These global responses are primarily governed by the deposition path, boundary conditions, and accumulated thermal contraction, and are therefore expected to be less sensitive to the exact cross-sectional shape than the local peak values.

Therefore, a virtual point is artificially set at the start coordinate, where (X3, Y3) is identical to (X1, Y1), and Z3 can take any arbitrary value. In this study, X3 is uniformly set to 1 across all local coordinate systems, thereby enabling the establishment of a local coordinate system for a single filament. By appending an additional command line “CSWPLA, n” subsequent to the WPLANE command line, the newly established local coordinate system can be assigned the serial number *n*.

With the local coordinate system established, any inclined printing path can be represented as a cuboid aligned with a single local direction. This treatment unifies the geometric representation of printing paths with different orientations of printing paths with different directions, thereby facilitating subsequent modeling of complex paths and the simulation processes.

#### 2.2.2. Geometric Modeling in the Local Coordinate System

Subsequent to the establishment of the local coordinate system, geometric modeling of the material filament is performed for every individual printing path. As previously described, the filament is modeled as a cuboid. Thus, the BLOCK command in APDL is employed herein for modeling purposes. In the command “BLOCK, X1, X2, Y1, Y2, Z1, Z2”, the six values represent the coordinates of the six faces of the cuboid in the current coordinate system, through which a cuboid is constructed. The start and end coordinates correspond to the midpoints of the bottom edges of the front and end faces of the cuboid, respectively. Specifically, when establishing the local coordinate system using the start and end coordinates, the absolute distance between these two points is calculated. After activating the local coordinate system, the BLOCK command is utilized for modeling, where X1 is set to 0, X2 is assigned the absolute distance, Y1 is set to 0, Y2 is assigned the layer height, and Z1 and Z2 (each being half of the line width) are set as opposite numbers to each other. At this point, the modeling of a single filament in the local coordinate system is completed.

**Figure 5:**
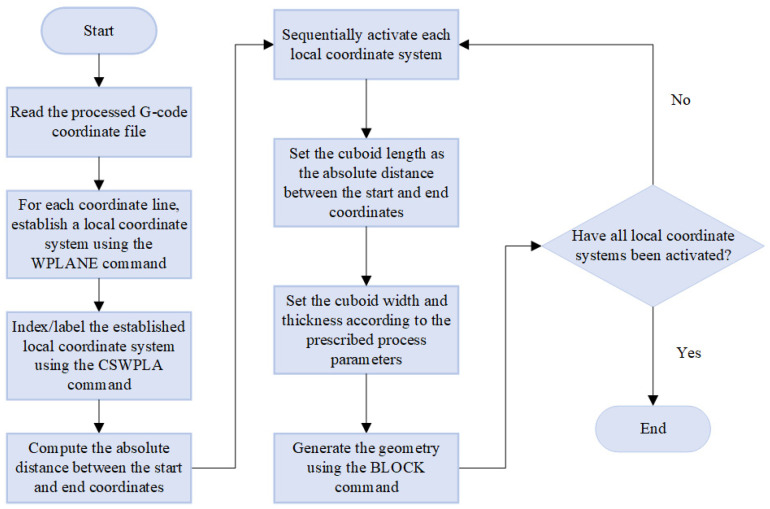
Workflow of Local-coordinate-based modeling.

**Figure 6:**
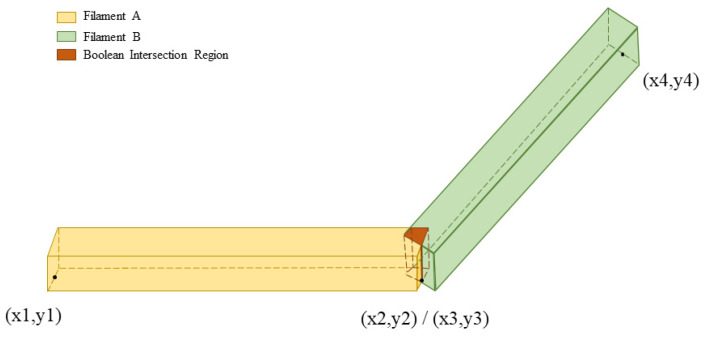
Boolean operation on overlapping filament segments.

By developing a C program to perform the modeling process, an overall geometric model consistent with the actual printing paths can be obtained. Figure 5 illustrates the detailed workflow implemented in the C program. Note that model overlapping occurs at the contact points of adjacent filaments, which is caused by the continuity of the G-code. For instance, within the same layer, Filament A traverses from (X1, Y1) to (X2, Y2), and Filament B traverses from (X3, Y3) to (X4, Y4). The coordinates (X2, Y2) and (X3, Y3) are identical, thus resulting in the overlapping of the corresponding filament models, as shown in Figure 6. Herein, Boolean operations are adopted to remove the overlapped volumes at filament intersections and generate new entities, thereby ensuring geometric continuity of the filament-level model. To improve robustness for fine-scale intersections, a geometric tolerance of 1 × 10^−6^ mm is used in Boolean operations. After Boolean processing, a geometry cleanup/merging step is performed using NUMMRG (merging coincident items) to eliminate possible duplicated entities and topological inconsistencies. In addition, the mesh is locally refined in the intersection regions to enhance meshing stability and avoid poor-quality elements. Compared with modeling strategies limited to paths parallel/perpendicular to the global coordinate system, the above procedure provides better geometric generality for complex toolpaths with inclined angles while maintaining numerical robustness.

### 2.3. Element Birth-Death Method Based on Element Center

EBDM has been commonly used to simulate the AM process by manipulating element birth or death. Its principle is as follows: all material parameters of element nodes are first multiplied by an extremely small value via a killing command, thereby excluding them from the thermal-mechanical coupled solution and defining them as unextruded filaments. Then, an activation command is adopted to restore the thermophysical parameters of the element nodes within the specified spatial coordinates, followed by the application of thermal loads to complete the simulation of extruded filaments.

**Figure 7:**
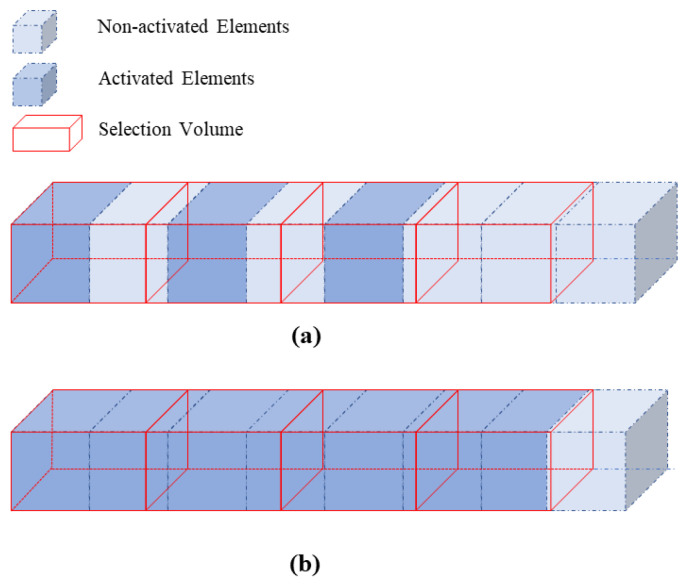
Two element-selection strategies: (**a**) selecting nodes within the spatial window and then selecting the elements attached to these nodes; (**b**) directly selecting elements whose centroids fall within the spatial window.

**Figure 8:**
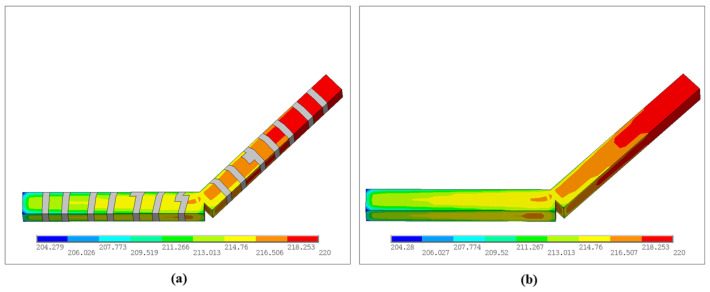
Results of the two element-selection strategies: (**a**) selecting nodes within the spatial window and then selecting the elements attached to these nodes; (**b**) selecting elements whose centroids fall within the spatial window.

Figure 7 illustrates two different element-selection strategies. As shown in Figure 7a, in the existing element activation methods, the command “NSEL, R, LOC, X, XMIN, XMAX” is executed in X, Y, Z directions to select nodes within a hexahedral space. Then, the command “ESLN, S, ALL” is executed to select elements attached to these nodes. This is because the activation command “EALIVE, ALL” can only be implemented through element selection. The advantage of this method is that it ensures the filaments are extruded in cubic volumes at each time step. However, it imposes strict requirements on mesh generation: the generated elements must be entirely enclosed within the hexahedral space. If an element has nodes distributed across two or more sequentially selected hexahedral spaces, it cannot be selected and will be excluded from the calculation, leading to the situation shown in Figure 8a. Therefore, to ensure the applicability of mesh generation, the previous research work in [16] still needed to further conduct segmented modeling for each filament after discretizing the global model into individual filaments.

In contrast, the G-code-driven modeling based on the local coordinate system does not require segmented modeling for single filaments. Additionally, Boolean operations at the joints of filaments would lead to difficulties in segmentation and complicated mesh generation. To address these issues, this study proposes a novel element selection method: the command “ESEL, R, CENT, X, XMIN, XMAX” is used in X, Y, Z directions to select elements whose centroids are within the hexahedral space, as shown in Figure 7b. Then, the “EALIVE, ALL” command is executed to activate the selected elements, followed by the “NSLE, S, ALL” command to select nodes attached to these activated elements for load application and subsequent simulation. Under this approach, although the “extruded” filament segment at each time step cannot be strictly constrained to a cubic volume, filament continuity is still preserved, as shown in Figure 8b. This method overcomes the constraint of being restricted to hexahedral elements. Instead, tetrahedral meshes demonstrate superior adaptability when dealing with complex geometries. The high level of flexibility in both mesh generation and element selection renders this method well-suited for models with complex printing paths.

### 2.4. Thermo-Mechanical Coupling Analysis Method

After the G-code-driven modeling and meshing, the EBDM is adopted to simulate the FDM process. A novel spatial coordinate selection method is proposed, which is adaptable to more complex models and mesh generation schemes. Then, the indirect coupling method is employed to accomplish the coupling between the temperature field and the mechanical field. Meanwhile, a C program is utilized to convert the command stream of the complex printing paths into a command stream for indirect coupling analysis, so as to solve the transient temperature field, stress field, and displacement field. This section will elaborate on the methodologies involved in this process in detail.

#### 2.4.1. Indirect Coupling Method

In the simulation of FDM processes, the solid element expands continuously via EBDM, accompanied by dynamic variations in heat sources and boundary conditions. Concurrently, material states and properties alter with temperature changes is involved. Therefore, the analysis of FDM processes, which requires accounting for temperature-dependent material property variations and nonlinear problems in the stacking of filament materials such as PLA and ABS, constitutes a typical nonlinear transient thermo-mechanical coupling problem.

ANSYS supports two solution approaches, namely direct coupling and indirect coupling. Direct coupling is suitable for thermo-mechanical coupling analyses with relatively simple temperature and stress fields. However, in FDM processes, the temperature and position of filaments change instantaneously, leading to a large number of degrees of freedom. Direct coupling would thus incur excessive computational costs. Therefore, indirect coupling is adopted for thermo-mechanical coupling analysis. Specifically, after solving the temperature field, the temperature values of each element node in the temperature field are extracted and applied as thermal loads to the stress field model to complete the solution of the stress field.

#### 2.4.2. Temperature Field Analysis

The following assumptions must be made prior to the temperature field analysis:

(1) The initial temperature of each element is uniformly distributed at the preset nozzle temperature upon activation, and only the activated elements undergo convective heat transfer with the ambient air;
(2) The specific heat capacity mutation method is employed to address the issue of latent heat of phase change. When the temperature of the printed part reaches the melting point, it is assumed that the temperature will not change until the latent heat is completely absorbed or released. The effect of latent heat is considered by defining the thermal enthalpy of the material at different temperatures, and it is postulated that the latent heat is released uniformly within the temperature range between the solidus and liquidus temperatures.

During the FDM process, the filament undergoes a phase transition cycle of solid state-molten state-solid state, and the evolution of the temperature field follows the law of energy conservation. Considering the temperature dependence of the material’s thermophysical parameters (i.e., density *ρ*, specific heat capacity *c*, thermal conductivity *k*) and the effect of latent heat of phase change, the governing equation for the transient temperature field is established as follows:

(1) $$\rho \left(T\right)c\left(T\right)\partial T/\partial t=\nabla \cdot \left[k\left(T\right)\nabla T\right]+\left(Q_{v}\right)$$

where *T* is the temperature; *t* is the time; $Q_{v}$ is the volumetric heat source term (i.e., the heating input from the nozzle, applied via nodal thermal loads in ANSYS); ∇ is the Hamiltonian operator, which characterizes the spatial gradient.

The latent heat of phase change is addressed using the specific heat capacity mutation method. By increasing the equivalent specific heat capacity within the phase transition temperature range (ABS: 180–220 °C), the latent heat is converted into an equivalent sensible heat:

(2) $$c_{eq}\left(T\right)=c\left(T\right)+\frac{L}{\Delta T_{phase}}$$

where $C_{eq}(T)$ is the equivalent specific heat capacity; *L* is the latent heat of phase change; ${\Delta T}_{phase}$ is the phase transition temperature range.

The boundary conditions of heat transfer during the FDM process involve three mechanisms: thermal convection and thermal conduction. Based on the printing environment and the contact characteristics with the build platform, the boundary conditions are defined as follows:

(1) Thermal convection boundary (printed part-air): The convective heat transfer between the surface of the printed part and the air obeys Newton’s law of cooling:

(3) $$-k\left(T\right)\frac{\partial T}{\partial n}{|}_{\Gamma_{1}}=h_{a}\left(T-T_{env}\right)$$

where $\Gamma_{1}$ is the convection boundary of heat flux; $h_{a}$ is the convective heat transfer coefficient (ranging from 7 to 15 W/(m^2^·K)); $T_{env}$ is the ambient temperature; n is the outward unit normal vector of the boundary.

(2) Thermal conduction boundary (printed part-build platform): The heat transfer through the contact between the printed part and the heated build platform, assuming zero contact thermal resistance at the interface:

(4) $$-k\left(T\right)\frac{\partial T}{\partial n}{|}_{\Gamma_{2}}=k_{s}\left(T-T_{s}\right)$$

where $\Gamma_{2}$ is the conduction boundary of heat flux; $k_{s}$ is the thermal conductivity of the build platform; $T_{s}$ is the preset temperature of the build platform (typically ranging from 80 to 120 °C for ABS material).

On the above basis, the Galerkin weighted residual method is employed for the discretization of the governing equation. Let the approximate solution of the temperature field be,

(5) Th=[N]{T}

where [N] is the shape function matrix and {T} is the nodal temperature vector. Substitute $T_{h}$ into the transient temperature field governing equation, integrate over the solution domain V, and derive the FE equation by combining integration by parts and the Gauss theorem:

(6) $$\left[C\left(T\right)\right]\frac{dT}{dt}+\left[K\left(T\right)\right]T=P$$

where [C(T)] is the heat capacity matrix; [K(T)] is the thermal conductivity matrix; {P} is the thermal load vector.

The layer-by-layer printing process is simulated using the EBDM via ANSYS. Given its high stability and suitability for nonlinear heat conduction problems, the backward Euler method is adopted for temporal discretization, which converts the derived FE equation into an algebraic equation system:

(7) $$\left(\frac{[C{]}^{n}}{\Delta t}+[K{]}^{n}\right)\{T{\}}^{n}=\frac{[C{]}^{n}}{\Delta t}\{T{\}}^{n-1}+\{P{\}}^{n}$$

where n denotes the current time step; n−1 denotes the previous time step; Δt is the time step size.

#### 2.4.3. Thermal-Mechanical Coupled Field Analysis

The following assumptions are made for solving the stress field:

(1) The material filament obeys the Von Mises yield criterion during deformation;
(2) The material filament follows the small deformation criterion in the printing process, i.e., the stress and strain of the filament exhibit a linear relationship;
(3) The material filament deposited along a single printing direction is continuous and isotropic internally;
(4) The deformation of the material filament occurring during the printing process is primarily temperature-dependent.

The evolution of the stress field follows the equilibrium equations and the thermoelastic-plastic constitutive relations. Considering the thermal strain induced by the temperature field, the thermo-mechanical coupling governing equations are established:

(8) ∇·[σ]=0

where [σ] is the stress tensor, which is solved by the constitutive equation $\left[\sigma \right]=[D](\left\{\varepsilon \right\}-{\left\{\varepsilon \right\}}_{T})$; {ε} is the total strain vector; ${\left\{\varepsilon \right\}}_{T}$ is the thermal strain vector; [D] is the elastic matrix.

The total strain vector is decomposed into elastic strain, plastic strain, and thermal strain:

(9) $$\{\varepsilon \}=\{\varepsilon {\}}_{e}+\{\varepsilon {\}}_{p}+\{\varepsilon {\}}_{T}$$

where ${\left\{\varepsilon \right\}}_{e}$ is the elastic strain obeying the generalized Hooke’s law; ${\left\{\varepsilon \right\}}_{p}$ is the plastic strain described by the Hill48 yield criterion.

The thermal strain ${\left\{\varepsilon \right\}}_{T}$ is defined as:

(10) $$\{\varepsilon {\}}_{T}=\left\{\alpha \left(T\right)\right\}\left(T-T_{0}\right)$$

where α(T) is the temperature-dependent coefficient of thermal expansion vector; $T_{0}$ is the reference temperature, which is set as the initial temperature (30 °C) in this study.

The approximate solution of the displacement field is assumed as

(11) {u}=[N]{U}

where {u} is the nodal displacement vector.

Substituting the above expression into the geometric equation

(12) $$\varepsilon =\nabla_{u}\left[N\right]U$$

where $\nabla_{u}$ is the displacement gradient operator, and combining it with the constitutive equation and equilibrium equation, the discretized governing equation is derived via the Galerkin method:

(13) $${\left[K\right]}_{s}\left\{U\right\}={\left\{F\right\}}_{T}$$

where ${\left[K\right]}_{s}$ is the stiffness matrix and ${\left\{F\right\}}_{T}$ is the thermal load vector, which are defined, respectively, as follows:

(14) $${\left[K\right]}_{s}=\int_{V}{\left[\nabla_{u}N\right]}^{T}\left[D\left(T\right)\right]\left[\nabla_{u}N\right]dV$$

(15) $$\{F{\}}_{T}=\int_{V}{\left[\nabla_{u}N\right]}^{T}\left[D\left(T\right)\right]\left[\{\varepsilon {\}}_{T}\right]dV$$

### 2.5. Hardware and Software Setup

To verify the correctness of the proposed method and theoretical formulation, a square plate with dimensions of 10 mm × 10 mm × 1 mm was selected as the benchmark geometry. Numerical investigations were conducted for the plate under different infill configurations, including triangular infill, hexagonal (honeycomb) infill, and hexagonal infill with an outer wall and a bottom plate, which are commonly adopted in practical printing to maintain surface integrity. The software and hardware used in both the FE simulations and the physical printing experiments are summarized in Table 1. The printing material was acrylonitrile–butadiene–styrene (ABS) filament, and its temperature-dependent material properties are listed in Table 2. After importing the CAD model into the slicing software, the key process parameters were set as shown in Table 3.

In the simulation, the bottom surface was modeled as a fixed boundary. To approximate this restrained condition experimentally, adhesive was applied to the build plate to enhance the attachment of the first deposited layer. Meanwhile, the build-plate temperature was limited to 35 °C to avoid overly strong adhesion that could hinder specimen removal and interfere with the subsequent warpage evaluation.

For the FE simulations, the temperature field was solved using SOLID70 elements. SOLID70 is an 8-node brick thermal element with one degree of freedom (temperature) per node, and is suitable for transient thermal analyses. Subsequently, for the stress field solution, the thermal element type was converted to SOLID185, which is also an 8-node brick structural element with three translational degrees of freedom per node, enabling thermo-mechanical stress–displacement computations.

## 3. Results

### 3.1. Square Plate with Triangular Infill

A square plate was first investigated using a triangular infill pattern. In the slicing software, the infill line spacing was set to 2 mm. The side length of each triangle was approximately 1.85 mm, and all triangles were equilateral. The total printing duration was 1 min 6 s. To balance computational efficiency with the model length scale, a mesh size of 0.2 mm was adopted for all simulations. A mesh-sensitivity assessment is provided in Section 3.2 based on a representative configuration, and the model was discretized using a sweeping meshing strategy.

**Figure 9:**
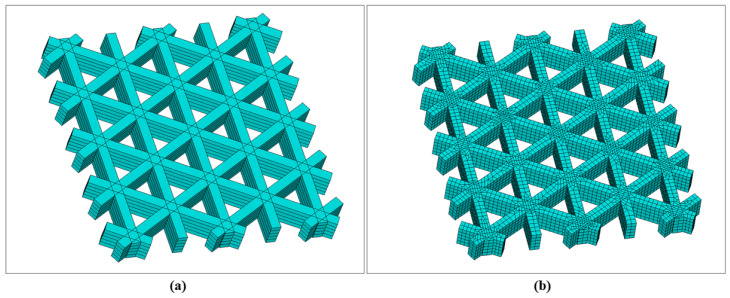
Filament-scale model and mesh of the triangular infill generated by the local coordinate discretization method: (**a**) geometric model; (**b**) FE mesh.

Figure 9 presents the geometry and meshing results of the triangular infill constructed via the local coordinate discretization approach. Figure 9a shows the filament-resolved solid model, in which the periodic triangular cells formed by interlaced printing paths are clearly identified, together with the intersection regions produced by Boolean operations. Figure 9b shows the corresponding FE mesh. The mesh remains continuous along the filament length direction following the paths and is locally refined in Boolean-intersection regions, thereby enabling subsequent transient thermal analysis to capture temperature gradients and the superposition of heat-affected zones. Hexahedral elements dominate the discretization; however, a small portion of hexahedral elements degenerates into tetrahedral elements near filament intersections to accommodate geometric complexity. By organizing both geometry and mesh along the printing path, this modeling strategy ensures that the spatial locations of stepwise material “activation” remain consistent with the actual deposition sequence, providing a robust basis for coupled thermal and mechanical analyses.

**Figure 10:**
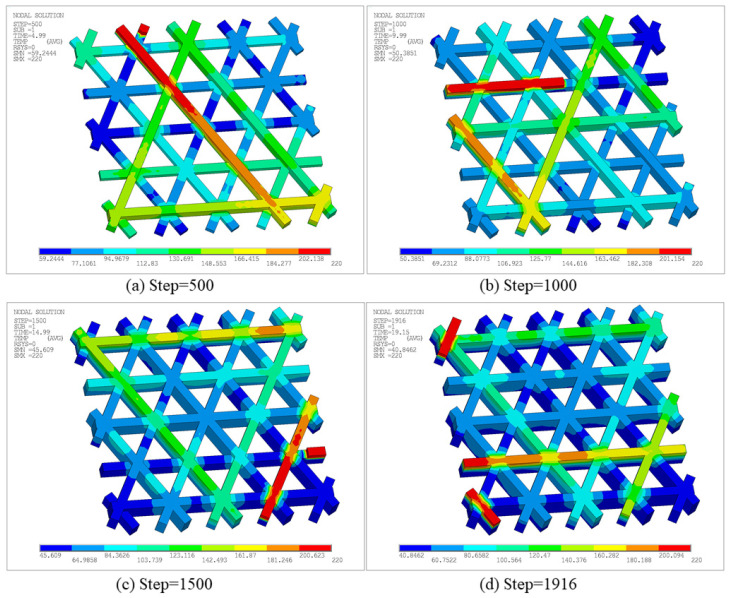
Temperature contours of the triangular infill at different time steps.

Figure 10 shows the temperature-field contours of the triangular infill at different time steps. The high-temperature region consistently accompanies the most recently activated material and forms a banded distribution along the printing paths. As the time steps advance, previously deposited regions gradually cool due to convective heat loss and internal conduction. Thus, the contour map transitions from warm to cool colors, exhibiting the typical transient thermal evolution in the FDM process. In terms of magnitude, the peak temperature at all steps remains close to the nozzle setpoint, approximately 220 °C. Meanwhile, the minimum temperature decreases progressively as the process proceeds, i.e., Solution Min (SMN) ≈ 58.77 °C at Step 500, SMN ≈ 54.12 °C at Step 1000, SMN ≈ 46.42 °C at Step 1500, and SMN ≈ 40.85 °C at the final Step 1916. This trend indicates that the accumulated heat is continuously released to the surroundings and the structure gradually returns toward ambient temperature. Notably, the Boolean-intersection regions and short turning segments in the triangular pattern are more prone to locally elevated temperature levels or steeper gradients. On one hand, these regions correspond to nozzle turning and closely spaced adjacent paths, which concentrate heat input over a short time. On the other hand, multiple paths intersect at nodes, yielding complex local heat capacity and conduction pathways and promoting overlap of heat-affected zones, such that the same location experiences pronounced reheating at later steps. In contrast, the temperature along a straight path typically exhibits a single heating and continuous cooling pattern, highlighting the dominant influence of the printing path on the local temperature field.

**Figure 11:**
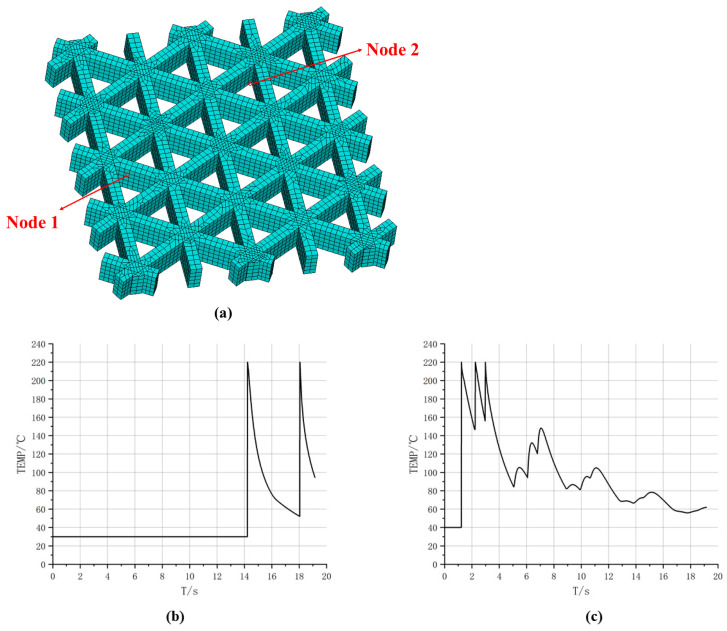
Temperature variations at selected nodes of the triangular infill: (**a**) FE mesh with node locations; (**b**) temperature variations at Node 1; (**c**) temperature variations at Node 2.

To further characterize location-dependent thermal histories in the time domain, Figure 11 reports temperature–time curves for two representative nodes, whose positions are displayed in Figure 11a. Both curves exhibit the rapid heating and decay cooling behavior induced by path-by-path deposition, yet they differ markedly in the timing and number of peaks. As shown in Figure 11b, the temperature at Node 1 remains at 30 °C from 0 s to 14.1 s, followed by a sharp rise at 14.1 s to a peak of 220 °C, and then gradually cools to approximately 55 °C. Till 18 s, Node 1 undergoes another rapid temperature increase to 220 °C, after which it cools to approximately 100 °C. This behavior suggests that the spatial location of Node 1 is not activated in the early stage. Instead, it is deposited later as the printing advances, and shortly after the first deposition it receives additional thermal input from neighboring paths, resulting in a “delayed activation and secondary reheating” pattern. In contrast, as shown in Figure 11c, Node 2 heats up to a peak of 220 °C at the beginning and then experiences multiple closely spaced peaks, followed by an overall slow decay. Several smaller reheating oscillations are observed during 6–12 s, and the temperature approaches a quasi-steady level of about 60 °C near 20 s. This curve indicates that Node 2 is located in an early-deposited region where node crossings exist. The initial deposition produces the first peak, while subsequent adjacent depositions lead to superposed thermal cycles that repeatedly elevate the cooling curve and form multiple pronounced peaks. Combined with the evolutions of high-temperature areas in Figure 10, these results indicate that the spatial thermal evolution of triangular infill is primarily driven by intersecting and overlapping printing paths, leading to non-uniform thermal strain and accumulated residual effects that directly impact subsequent stress analysis.

**Figure 12:**
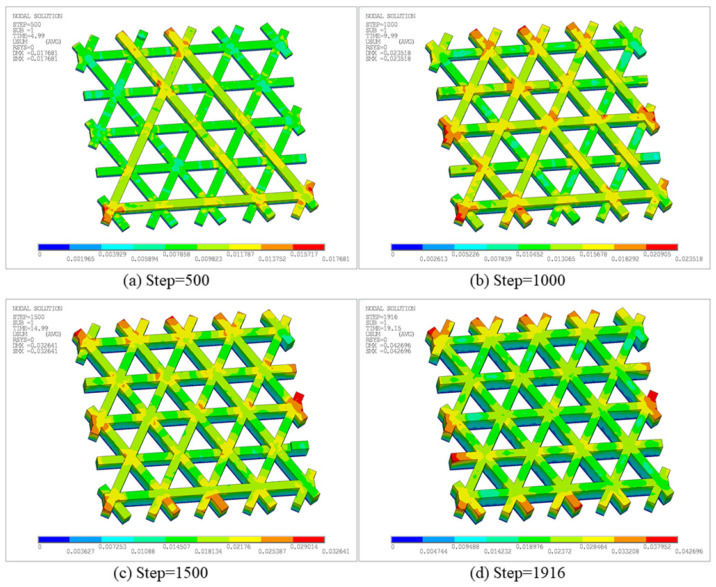
Displacement contours of the triangular infill at different time steps.

Figure 12 presents the total displacement contours of the triangular infill at different time steps. The displacement field evolves from localized accumulation to a more global distribution. At Step 500, a noticeable deformation occurs, with a maximum total displacement, i.e., Solution Max (SMX), of 1.7681 × 10^−2^ mm, as shown in Figure 12a. High-displacement regions are mainly located at free outer boundaries and filament intersections, indicating that early warping caused by heat input and cooling contraction is first released in areas with relatively weaker constraints and geometric discontinuities. As printing proceeds, the maximum displacement increases to 2.3518 × 10^−2^ mm at Step 1000, as shown in Figure 12b. The high-displacement region extends along longer connected paths and boundary directions. This reveals that, under repeated thermal cycling, the internal filling structure distributes local shrinkage into larger-scale deformations. At Step 1500, the maximum displacement further increases to 3.2641 × 10^−2^ mm, as shown in Figure 12c. The mid-to-high displacement region expands significantly, implying that residual deformation accumulates with continued thermal cycling. Meanwhile, the displacement gradient in the overlapping areas of materials becomes more pronounced, reflecting the more complex thermal and mechanical constraints at these locations. This is primarily due to the local overlap of the heat-affected regions caused by the proximity of adjacent printing paths, which promotes structural deformation. Finally, at Step 1916, the maximum total displacement reaches 4.2696 × 10^−2^ mm, as shown in Figure 12d. The overall displacement level is considerably higher than in early stages, and the highest displacement remains concentrated near the free outer boundary, indicating that late-stage deformation in this open lattice structure is governed by boundary freedom and non-uniform global shrinkage. In summary, the deformation of the triangular infill does not grow linearly but instead accumulates continuously with the deposition sequence and the number of local thermal cycles. Free outer boundaries and Boolean-intersection regions constitute key locations of displacement concentration, consistent with the more intensive heat input at the turning and connecting points of the printing paths.

**Figure 13:**
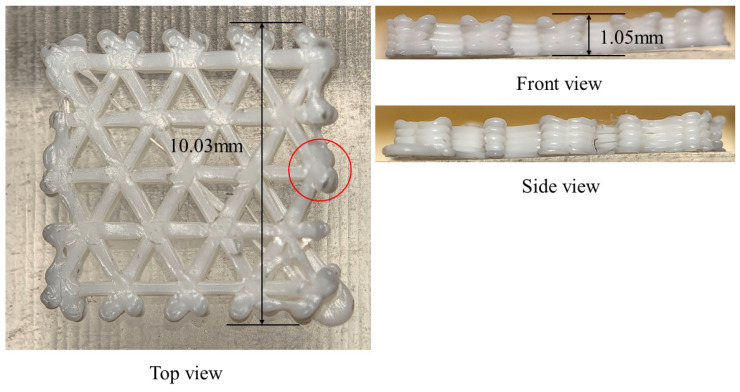
Three views of the real printed triangular-infill specimen.

As shown in Figure 13, the triangular-infill specimen was printed and its final warpage was measured after cooling to room temperature. The printed part exhibits visible out-of-plane deformation near the free outer boundary, which is consistent with the simulation result showing that the largest displacement is concentrated near the outer edges and filament-intersection regions. Quantitatively, the simulated final maximum displacement is 4.2696 × 10^−2^ mm, whereas the experimentally measured warpage is 5 × 10^−2^ mm (as marked in Figure 13), giving a relative difference of 14.6%. Although the absolute value is not identical, the experiment reproduces the same deformation tendency and dominant warpage location, indicating that the proposed model can reasonably capture the warpage evolution of the triangular infill structure.

**Figure 14:**
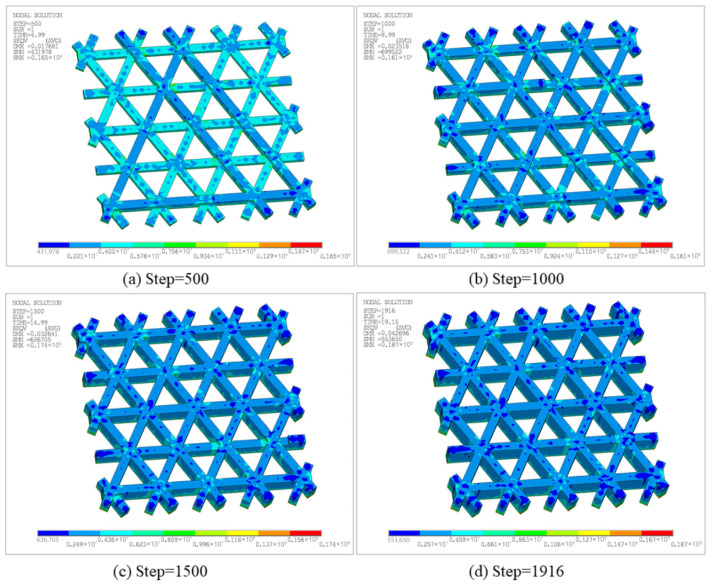
Equivalent stress (SEQV) contours of the triangular infill at different time steps.

Figure 14 presents the equivalent stress at different time steps, characterizing residual stress levels and their spatial distribution under the combined effects of non-uniform thermal strain and mechanical constraints. Note that a fully fixed boundary condition was applied to the entire bottom surface. Therefore, the maximum stress is concentrated in the fixed bottom region, particularly near bottom boundaries and locations where the printing direction changes and constraints vary abruptly. At Step 500, the equivalent stress is generally at a low-to-moderate level, with a maximum of 1.65 × 10^7^ Pa and a minimum of 4.3198 × 10^5^ Pa, as shown in Figure 14a. This indicates that even in the early stage, temperature gradients between newly deposited hot regions and cooled regions cause shrinkage mismatch and produce stress concentrations under bottom constraints. As the step increases to 1000, the maximum equivalent stress is 1.61 × 10^7^ Pa, as shown in Figure 14b, with a distribution similar to the early stage. High-stress regions remain mainly near boundaries and node intersections, attributable to faster cooling at the outer edge and the difficulty of accommodating thermal shrinkage at stiffness discontinuities. At Step 1500, the maximum equivalent stress increases to 1.74 × 10^7^ Pa, as shown in Figure 14c, and the area of moderate stress expands, indicating broader accumulation of thermally induced strain incompatibility under repeated thermal cycling. Finally, at Step 1916, the maximum equivalent stress further increases to 1.87 × 10^7^ Pa, as shown in Figure 14d, with a minimum of approximately 5.5365 × 10^5^ Pa, representing the highest overall stress level during the FDM process. In general, the maximum equivalent stress increases gradually during printing, whereas high-stress regions remain as discrete points rather than developing into a continuous high-stress band, which can be attributed to the deformation-release capability of the triangular lattice structure. Taken together with Figure 12 and Figure 14, the results demonstrate the coexistence of accumulated displacement and progressively intensified stress near the fixed bottom surface, reflecting the coupled effects of printing temperature, geometry, and boundary constraints on residual stress evolution.

### 3.2. Square Plate with Hexagonal Infill

#### 3.2.1. Simulation Results of Square Plate with Hexagonal Infill

**Figure 15:**
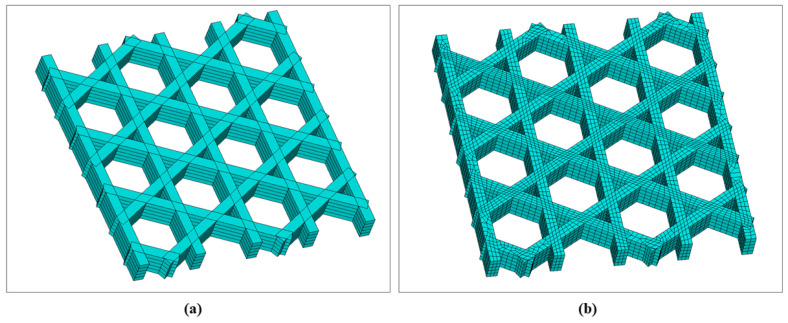
Filament-scale model and mesh of the hexagonal infill generated by the local coordinate discretization method: (**a**) geometric model; (**b**) FE mesh.

The square plate was further investigated using a hexagonal (honeycomb) infill pattern. In the slicing software, the infill line spacing was set to 2.4 mm, and the hexagon side length was approximately 1.73 mm. The total printing duration was 56 s. The mesh size was set to 0.2 mm, and a sweeping meshing strategy was employed. For the hexagonal infill configuration, the filament-scale geometry was first reconstructed using the proposed local coordinate discretization approach, followed by FE meshing, as shown in Figure 15. Figure 15a illustrates the periodic hexagonal topology formed by interlaced paths in the plane. Figure 15b presents the corresponding mesh, where the elements exhibit good alignment along the filament direction and maintain continuity across Boolean-intersection regions.

**Figure 16:**
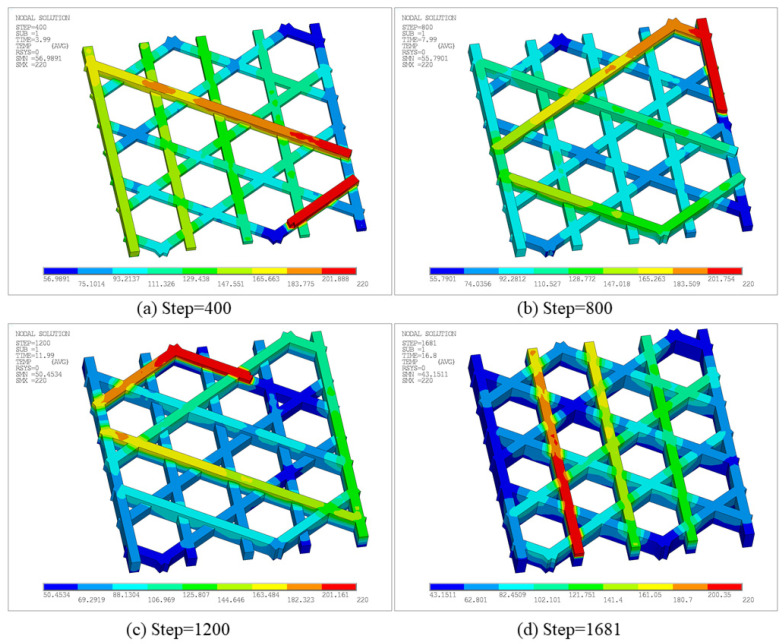
Temperature contours of the hexagonal infill at different time steps.

Figure 16 presents the temperature contours at different time steps. At each step, the maximum temperature remains concentrated near the most recently deposited path, resulting in a high-temperature band consistent with the current nozzle motion. In contrast, previously deposited regions cool progressively under the combined effects of heat conduction and surface convection, resulting in a temperature gradient that decays outward from the newly extruded material.

**Figure 17:**
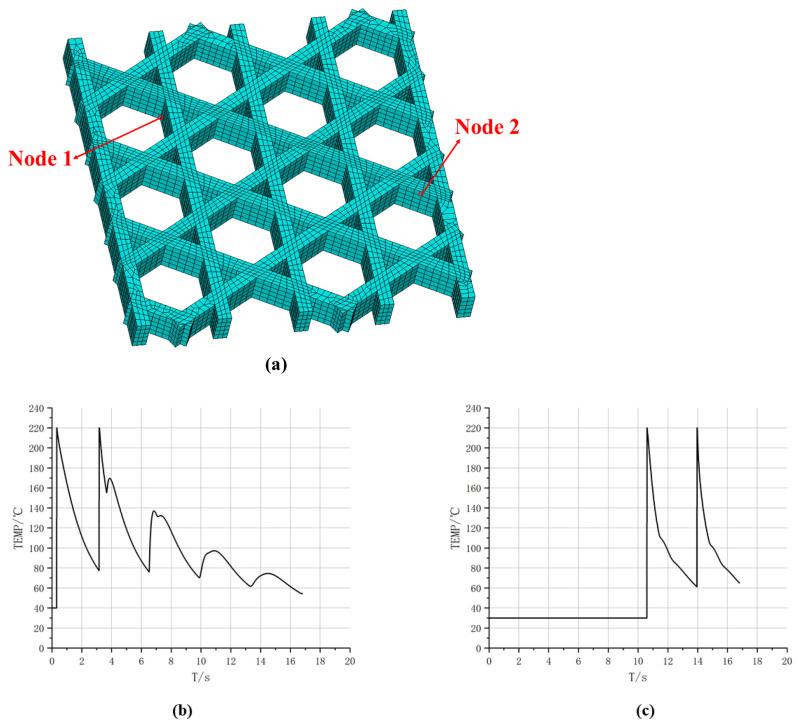
Temperature variations at selected nodes of the hexagonal infill: (**a**) FE mesh with node locations; (**b**) temperature variations at Node 1; (**c**) temperature variations at Node 2.

To further reveal location-dependent differences in local thermal histories, Figure 17 plots temperature–time curves at two representative nodes. The temperature at Node 1 exhibits two major peaks with an overall decaying trend. Specifically, the temperature rises sharply to a high peak when the nozzle first passes and deposits material, then cools gradually as the nozzle moves away. During subsequent deposition near a path-intersection region, the node heats to a second peak. Thereafter, smaller reheating fluctuations occur, with increasingly larger time intervals between peaks, indicating that reheating effects diminish as deposition proceeds farther away and the global temperature level decreases. Node 2 remains close to ambient temperature for an extended early period, and only rises markedly when later time steps bring the nozzle to its location. A second peak appears upon a subsequent pass, followed by cooling. This long low-temperature stage followed by two prominent peaks suggests that the corresponding material segment is deposited later, and its thermal input is dominated by direct nozzle passage. Overall, Figure 17 demonstrates that the number of heating events, peak timing, and cooling rate vary significantly across the hexagonal infill, fundamentally governed by spatial proximity among toolpaths and deposition time, thereby providing a thermal explanation for subsequent non-uniform displacement and residual-stress evolution.

**Figure 18:**
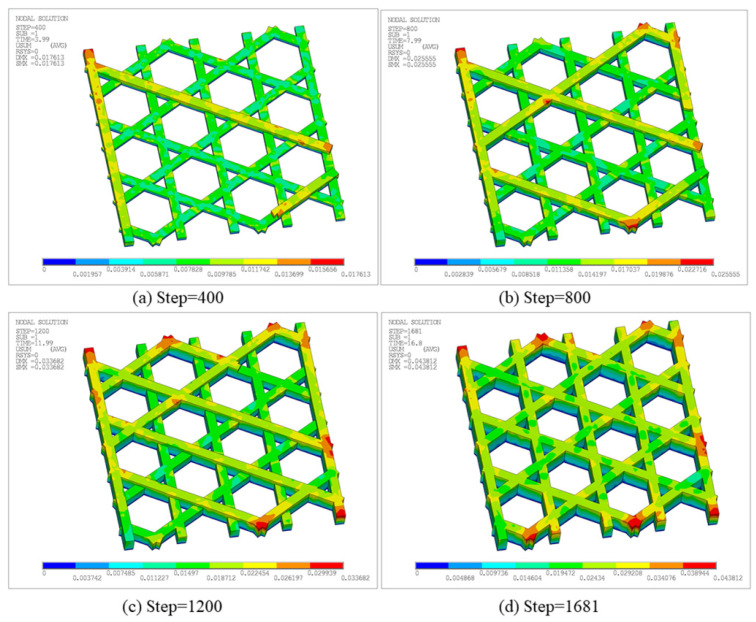
Displacement contours of the hexagonal infill at different time steps.

Figure 18 presents the total displacement contours at different time steps. As deposition proceeds, the maximum displacement increases monotonically: SMX = 1.7613 × 10^−2^ mm at Step 400, increasing to 2.5555 × 10^−2^ mm at Step 800, and reaching 3.3682 × 10^−2^ mm and 4.3812 × 10^−2^ mm at Step 1200 and the final Step 1681, respectively. High-displacement regions are primarily concentrated at the free outer boundaries and near adjacent filament junctions, and expand outward with continued printing. This indicates that thermal cycling induces non-uniform shrinkage and boundary warpage in the open lattice, while junction regions exhibit more pronounced displacement contrasts due to connectivity-induced constraints.

**Figure 19:**
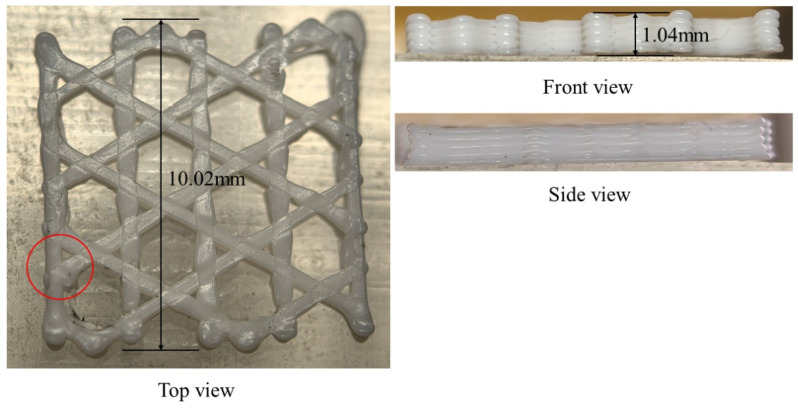
Three views of the real printed hexagonal-infill specimen.

As show in Figure 19, for the hexagonal-infill configuration, the printed specimen also shows noticeable warpage after printing, mainly along the free outer contour, which agrees well with the displacement pattern predicted by the simulation. The numerical model gives a final maximum displacement of 4.3812 × 10^−2^ mm (as marked in Figure 19), while the experimentally measured warpage is 4 × 10^−2^ mm, corresponding to a relative difference of 9.5%. Compared with the triangular infill, the hexagonal topology exhibits a similar deformation mode but a slightly different spatial distribution due to its distinct path connectivity and local constraint conditions. The consistency between the measured and simulated warpage supports the predictive capability of the filament-level thermo-mechanical model for open lattice structures.

**Figure 20:**
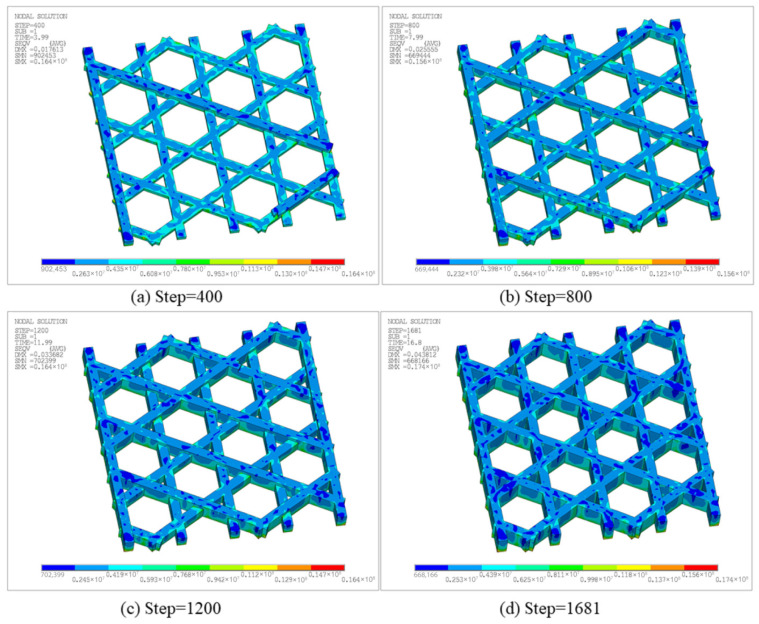
SEQV contours of the hexagonal infill at different time steps.

Figure 20 further presents the equivalent stress contours at the corresponding time steps. Overall, stress concentrations appear predominantly as discrete points, preferentially located near regions in contact with the build plate. The maximum equivalent stress at Step 400, 800, 1200, and 1681 reaches 1.64 × 10^7^ Pa, 1.56 × 10^7^ Pa, 1.64 × 10^7^ Pa, and 1.74 × 10^7^ Pa, respectively, indicating an overall accumulation trend of residual stress during printing. Peak locations remain relatively stable, primarily due to larger temperature gradients caused by stronger heat dissipation at outer edges and the difficulty of accommodating thermal shrinkage at stiffness-discontinuity nodes. Meanwhile, because the hexagonal lattice allows partial deformation release, high-stress regions do not develop into large continuous bands. Combined with Figure 18 and Figure 20, the structure exhibits coupled behavior, characterized by gradually intensifying displacement accumulation and stress concentration areas.

#### 3.2.2. Mesh Sensitivity Study

**Figure 21:**
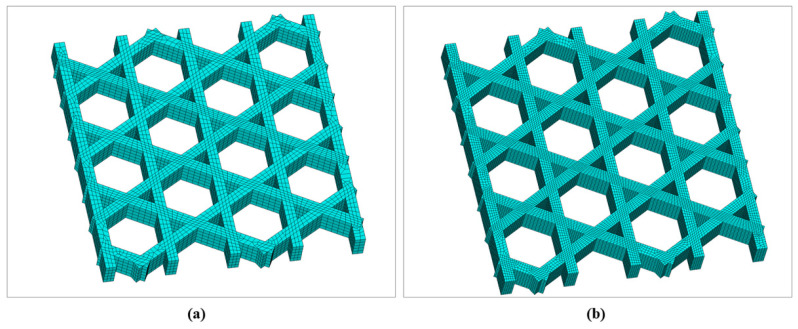
Comparison of hexagonal infill under two mesh sizes: (**a**) mesh size 0.2 mm; (**b**) mesh size 0.1 mm.

The baseline discretisation in this work adopts a mesh size of 0.2 mm, which is comparable to the layer thickness and therefore results in one element through the thickness, as shown in Figure 21. To assess the influence of through-thickness resolution on the predicted thermo-mechanical responses, an additional simulation was conducted for the hexagonal infill case using a refined mesh size of 0.1 mm, providing two elements through the thickness.

**Figure 22:**
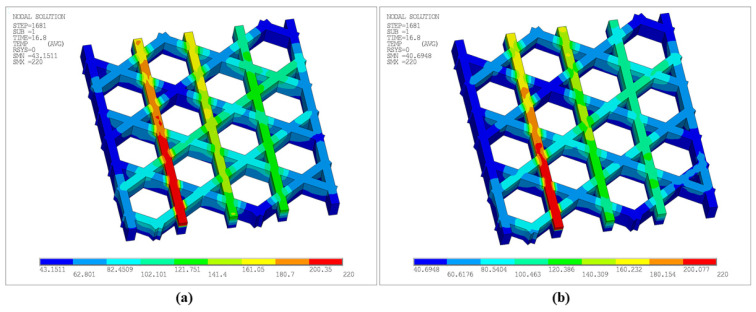
Comparison of temperature contours predicted using different mesh sizes: (**a**) mesh size 0.2 mm; (**b**) mesh size 0.1 mm.

Figure 22 compares the temperature contours at the end of printing obtained using the two mesh sizes. The overall thermal-field characteristics are consistent: the high-temperature zone remains concentrated near the most recently deposited paths and follows the toolpath sequence, indicating that the global heat-transfer behaviour is not qualitatively altered by mesh refinement. The mesh sensitivity is further quantified in Table 4 by comparing the end-of-printing maximum warpage and maximum von Mises stress. For the baseline 0.2 mm mesh, the final maximum displacement is 4.3812 × 10^−2^ mm, whereas the refined 0.1 mm mesh yields 3.9194 × 10^−2^ mm, corresponding to a difference of approximately 10.5%. For the maximum equivalent stress, the baseline mesh yields 1.74 × 10^7^ Pa, while the refined mesh yields 1.89 × 10^7^ Pa (difference ≈ 8.6%).

Overall, the refined mesh mainly affects local peak quantities (especially stress concentrations), while the global distributions and the comparative conclusions drawn from Case 2 remain unchanged. Considering the computational cost of filament-resolved transient thermo-mechanical simulations, the 0.2 mm mesh is retained for the remaining parametric analyses.

### 3.3. Square Plate with Hexagonal Infill, Sidewalls and Bottom Base

In practical printing setups, sidewalls and a bottom plate are often required to maintain surface integrity. Accordingly, the square plate was investigated with hexagonal infill while incorporating a continuous outer wall and a bottom layer. The infill line spacing remained 2.4 mm, and the hexagon side length was 1.73 mm. The outer-wall width was set to 0.4 mm (corresponding to one wall line), and the bottom-layer thickness was 0.2 mm (corresponding to one bottom layer). The total printing duration was 1 min 21 s. The mesh size was set to 0.2 mm, and a free meshing strategy was adopted.

**Figure 23:**
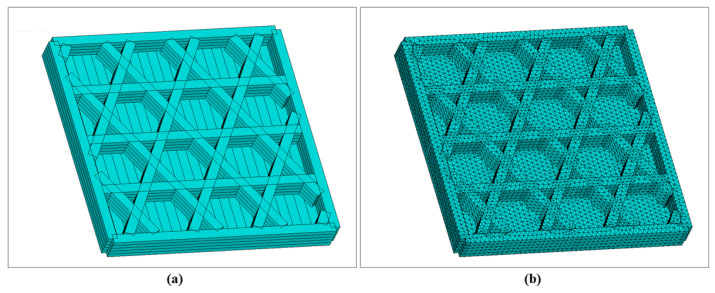
Filament-scale model and mesh of the hexagonal infill with a sidewall and bottom plate generated by the local coordinate discretization method: (**a**) geometric model; (**b**) FE mesh.

Figure 23 shows the reconstructed geometry and corresponding mesh generated by the local coordinate discretization approach. As shown in Figure 23a, the introduction of the bottom plate and sidewall significantly alters the heat-transfer boundary conditions. Specifically, the bottom plate provides a continuous conduction pathway, while the sidewall increases circumferential material accumulation and local thermal mass. Correspondingly, the mesh in Figure 23b achieves close geometric conformity at wall corners, filament intersections, and the connection regions between the bottom plate and vertical wall, establishing a basis for capturing temperature gradients and residual stresses in these critical locations.

**Figure 24:**
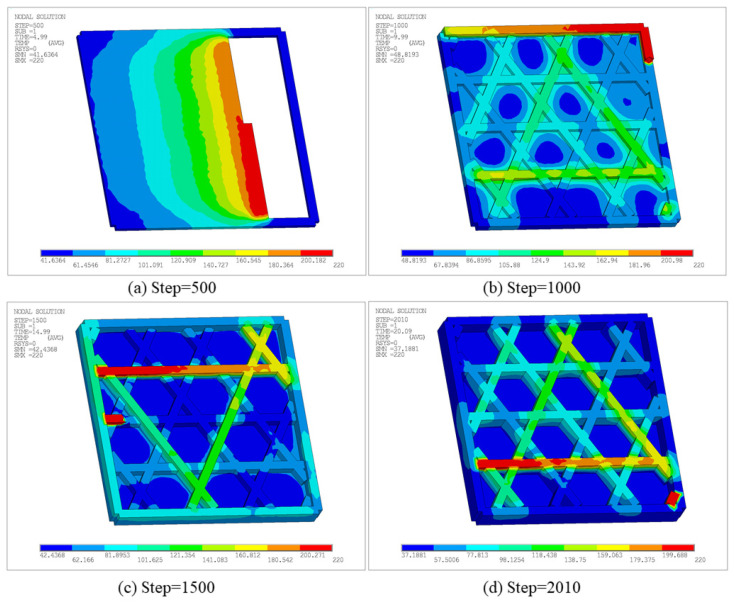
Temperature contours of the hexagonal infill with a sidewall and bottom plate at different time steps.

Figure 24 presents temperature contours at different time steps to characterize the transient thermal evolution. The peak temperature remains near the nozzle setpoint, indicating that heat input is still primarily carried by newly activated segments. However, compared with the open infill case, the low-temperature level is more strongly influenced by heat conduction through the bottom plate and the enclosure effect of the sidewall, thus cooling is not strictly monotonic. The minimum temperature is approximately 41.64 °C at Step 500, increases to 48.82 °C at Step 1000, suggesting an elevated thermal baseline due to heat feedback from the bottom plate and sidewall. Then, as the volume of the part and the heat transfer area increased, the cooling time in some areas was prolonged. Therefore, the minimum temperature decreases to 42.44 °C and 37.19 °C at Step 1500 and the final Step 2010, respectively. In addition, due to the path turning and small path spacing, short-term heat input is concentrated, resulting in local temperature accumulation not only along the infill paths, but also near the closed sections of the sidewalls and infill connections.

**Figure 25:**
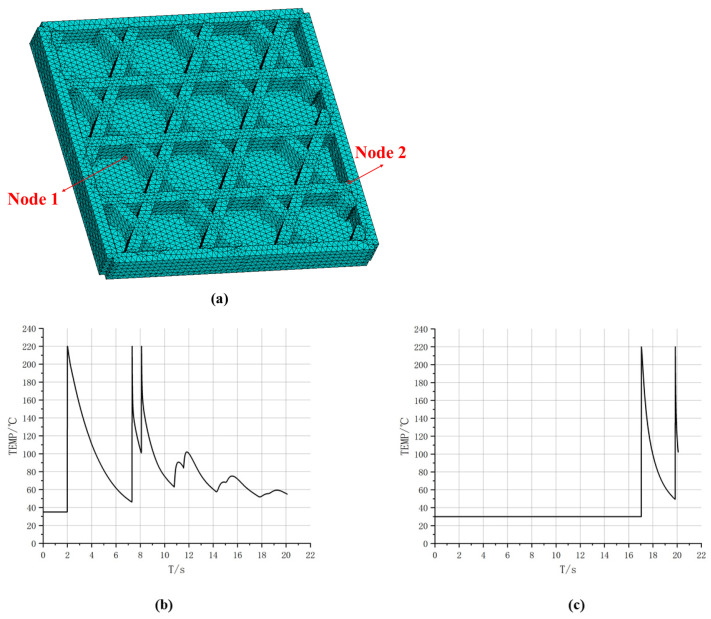
Temperature variations at selected nodes of the hexagonal infilled plate: (**a**) FE mesh with node locations; (**b**) temperature variations at Node 1; (**c**) temperature variations at Node 2.

Furthermore, Figure 25 compares temperature–time curves at two representative nodes. For Node 1, one peak appears at an earlier stage, followed by two similar peaks after a time interval. The first peak is associated with bottom-plate deposition, whereas the latter two arise from deposition of intersecting filaments. Subsequently, due to repeated thermal cycling in adjacent regions, the lower bound of the cooling phase remains at a relatively high level. In contrast, the temperature of Node 2 remains close to the air temperature for a longer period of time, only experiencing a sharp rise and rapid cooling in a short period of time, which represents the typical thermal load of the later printed area. These results indicate that after introducing the sidewall and bottom plate, the temperature evolution is not only influenced by the deposition sequence, but also by the thermal conduction paths introduced by the continuous solid, resulting in significant differences in reheating intensity and cooling rate.

**Figure 26:**
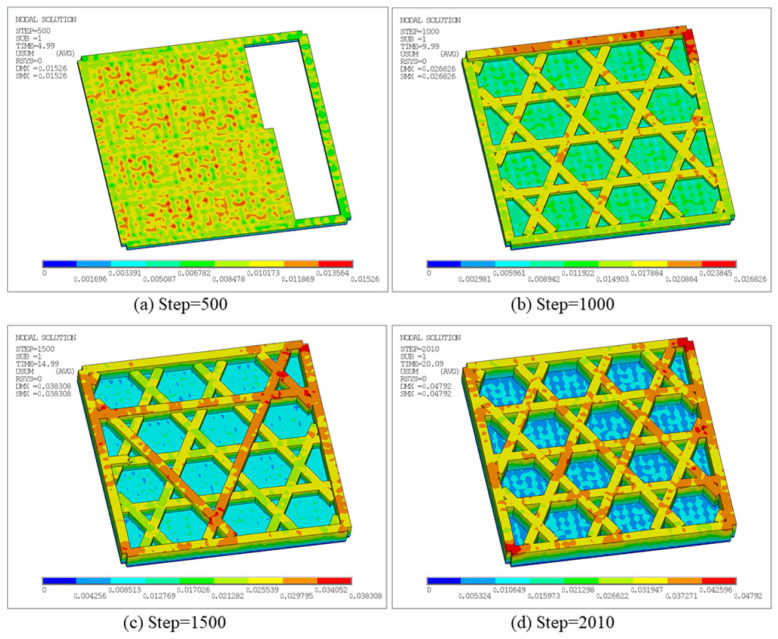
Displacement contours of the hexagonal infill with a sidewall and bottom plate at different time steps.

Figure 26 shows the displacement distribution at different time steps, characterizing warpage evolution under the additional constraints introduced by the sidewall and bottom plate. At Step 500, deformation remains low overall, with a maximum displacement of 1.526 × 10^−2^ mm. Displacements are discretely distributed along the sidewall edge and local filament junctions, reflecting the incompatible shrinkage caused by rapid cooling of the deposited materials in different regions. At Step 1000, the maximum displacement increases to 2.6826 × 10^−2^ mm, and the high-displacement region transitions from a discrete distribution to a concentrated distribution along the outer contour. At Step 1500 and the final Step 2010, the maximum displacement further increases to 3.8308 × 10^−2^ mm and 4.792 × 10^−2^ mm, with a broader affected area. Nevertheless, deformation remains constrained by the closed boundary imposed by the sidewall and the internal diagonal support, and thus concentrates mainly along the outer frame and internal infill.

**Figure 27:**
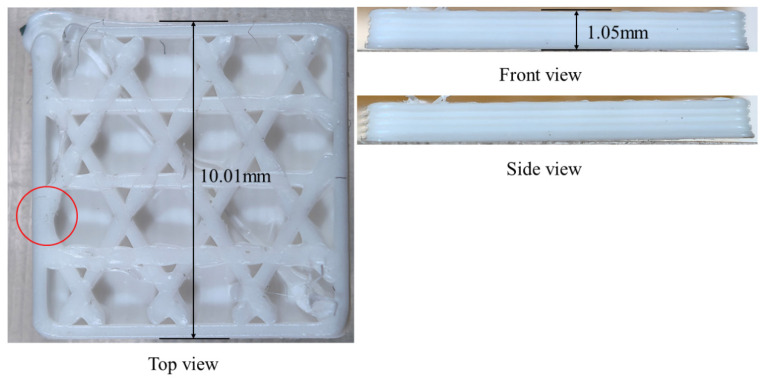
Three views of the real printed hexagonal infill with a sidewall and bottom specimen.

After introducing the sidewalls and bottom base, the printed specimen still exhibits measurable warpage, but the deformation is more strongly constrained by the closed boundary and the additional bottom support. This agrees with the simulation, in which the displacement remains concentrated along the outer frame and the internal infill rather than freely spreading over the whole plate. The simulated final maximum displacement is 4.792 × 10^−2^ mm, whereas the experimentally measured warpage is 5 × 10^−2^ mm (as marked in Figure 27), yielding a relative difference of 4.2%. The experiment therefore confirms that the addition of sidewalls and a bottom base significantly changes the warpage mode and boundary constraint effect, and the simulation captures this trend correctly.

**Figure 28:**
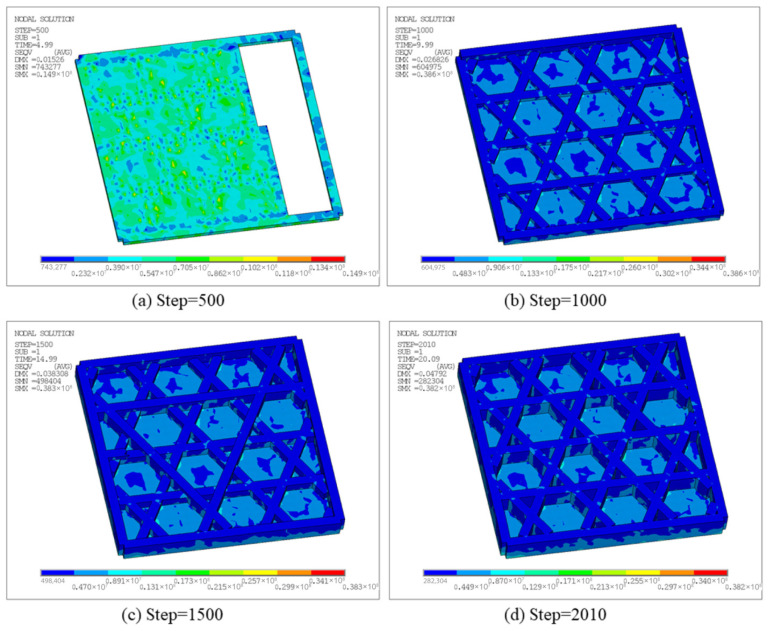
SEQV contours of the hexagonal infill with a sidewall and bottom plate at different time steps.

Figure 28 presents the equivalent stress contours at different time steps, reflecting the residual stress level and spatial distribution under the combined effects of non-uniform thermal strain and constraints. At Step 500, the maximum equivalent stress is 1.49 × 10^7^ Pa, with stress uniformly distributed on the bottom surface due to the constraint of the bottom plate. At Step 1000, the maximum increases to 3.86 × 10^7^ Pa, with stress concentrated in the contact regions between the infill and the bottom plate. At Step 1500 and Step 2010, the maximum equivalent stress is 3.83 × 10^7^ Pa and 3.82 × 10^7^ Pa, respectively, indicating that the overall stress level tends to stabilize and the contour patterns remain similar to those at Step 1000.

## 4. Discussion

The three case studies indicate that, although the peak temperature remains close to the nozzle setpoint in all configurations, the subsequent thermal histories, warpage patterns, and stress distributions are strongly affected by the path topology and boundary continuity. Table 5 presents a quantitative comparison of the simulated displacement, experimental warpage, relative difference, and maximum equivalent stress for different infill configurations. In the triangular and open hexagonal infills, the high-temperature region consistently follows the newly deposited path, while the previously deposited material cools progressively, indicating that the transient thermal field is mainly governed by the deposition sequence and local path adjacency. In contrast, after the sidewalls and bottom base are introduced, the temperature evolution is additionally influenced by the continuous solid boundary, which provides a more effective conduction pathway and increases the local thermal mass. As a result, the thermal baseline is elevated and reheating effects become more pronounced in regions near the wall–infill connections and the bottom-supported zones.

From the deformation perspective, all three configurations exhibit cumulative warpage growth with printing progression, confirming that residual deformation in FDM is not generated instantaneously but develops through repeated thermal cycling and non-uniform shrinkage. However, the mechanism of deformation accumulation differs among the three structures. For the triangular infill, the displacement concentration is strongly associated with free outer boundaries and filament intersections, where turning paths and repeated local reheating intensify the mismatch of thermal contraction. The open hexagonal infill shows a similar global deformation mode, but its path connectivity is more regular and the local constraint transfer is smoother, which leads to a slightly different spatial distribution of warpage. After the sidewalls and bottom base are added, the overall maximum displacement becomes the largest among the three cases, but the deformation no longer spreads as freely as in the open lattices. Instead, it is redistributed and constrained along the outer frame and internal support regions. This result suggests that adding enclosing boundaries does not necessarily reduce the maximum warpage; rather, it changes the manner in which thermally induced shrinkage is accommodated. In other words, geometric closure enhances structural continuity, but at the same time introduces stronger incompatibility of thermal contraction between the infill and the surrounding solid frame.

The experimental warpage measurements further support the above deformation trends. For the triangular infill, the simulated final maximum displacement is 4.2696 × 10^−2^ mm, compared with an experimentally measured warpage of 5 × 10^−2^ mm. For the open hexagonal infill, the corresponding values are 4.3812 × 10^−2^ mm and 4 × 10^−2^ mm. For the hexagonal infill with sidewalls and bottom base, the simulation predicts 4.792 × 10^−2^ mm, while the measured value is 5 × 10^−2^ mm. Although some differences remain in the absolute values, the experiments reproduce the same topology-dependent warpage tendency and confirm the dominant deformation locations predicted by the simulations. In particular, both the simulation and experiment indicate that the addition of sidewalls and a bottom base changes the deformation mode from a relatively open boundary-driven response to a more frame-constrained warpage pattern. Therefore, the present model is able to capture the main deformation characteristics associated with different infill and boundary layouts.

A clearer difference among the three configurations is observed in the stress field. The triangular and open hexagonal infills both exhibit relatively discrete stress concentrations, mainly near the bottom constrained region and local stiffness-discontinuity points, while their final peak equivalent stresses remain at the same order of magnitude. This indicates that open lattice topologies can partially release thermally induced deformation through their internal geometric compliance, thereby preventing the development of broad continuous high-stress bands. By contrast, the model with sidewalls and bottom base shows a much higher stress level, and the stress concentrates preferentially in the contact regions between the infill and the bottom-supported continuous solid. This demonstrates that the additional wall/base structure enhances thermal conduction and geometric integrity, but also significantly increases mechanical restraint during cooling. Therefore, a trade-off emerges: open lattices are more favorable for stress relaxation, whereas closed boundary configurations are beneficial for structural integrity and manufacturability but are more prone to stress accumulation.

These comparisons provide several engineering implications for FDM lightweight-structure design. If the primary objective is to reduce residual stress concentration, open lattice topologies are advantageous because they provide more deformation-release capability. If the design requires improved contour integrity or bottom-surface continuity, the addition of sidewalls and bottom layers is reasonable, but the associated increase in stress concentration should be considered in advance. Moreover, the present results suggest that evaluating infill configurations only by geometric stiffness or printability is insufficient; the coupled effects of toolpath connectivity, local reheating, boundary continuity, and shrinkage constraint must also be considered in thermo-mechanical design. The above trends are also consistent with previous studies showing that warpage and residual stress in FDM are highly sensitive to path arrangement, infill strategy, and structural constraints. Existing thermo-structural simulations based on element birth–death strategies have similarly reported that deposition sequence and filling strategy dominate the spatial evolution of thermal gradients and deformation, while toolpath-informed modeling improves the physical fidelity of process simulation [15,16,17]. In addition, recent path-driven and G-code-based modeling studies have emphasized that representing the as-manufactured filament layout is important for capturing local geometry-dependent responses that are difficult to reproduce using idealized continuum models [19,20,21,22,23]. Therefore, the present results further support the view that toolpath-aware filament-level modeling is particularly valuable when comparing lightweight structures with different internal topologies.

## 5. Limitations

- Equivalent isotropic constitutive assumption for each filament.

Each deposited filament is modeled using an equivalent isotropic constitutive description as a first-order approximation. This treatment is suitable for the present comparative analysis of topology-dependent thermal history, warpage evolution, and residual-stress distribution. However, it does not explicitly account for the direction-dependent behavior induced by raster orientation, interlayer bonding, and layer-wise deposition. Therefore, the current model is more suitable for comparative thermo-mechanical analysis than for accurate prediction of anisotropic mechanical properties of printed parts.

- Simplified filament geometry.

The deposited filament is simplified as a cuboid cross-section rather than the actual flattened or approximately elliptical bead geometry. This simplification improves the robustness of Boolean operations, meshing, and element activation, while preserving the nominal deposited volume defined by the line width and layer height. Nevertheless, the exact bead geometry may influence local heat dissipation and stress concentration, especially near filament intersections, and may therefore affect local peak responses.

- Simplified thermal boundary conditions.

Although the revised manuscript includes the ambient environment, cooling condition, and build-plate-related settings, the actual printing process may still involve more complex heat-transfer mechanisms, including local airflow disturbance, imperfect thermal contact with the build plate, and time-dependent environmental interactions. Moreover, the build-plate temperature adopted in this study (35 °C) was chosen to match the specific experimental setup used for validation, as a compromise between maintaining bottom-surface restraint during deposition and ensuring practical specimen removability after printing. As a result, the absolute temperature predictions and the corresponding warpage values may differ from those under more realistic thermal boundary conditions or under the higher build-plate temperatures more commonly used in standard ABS printing.

- Limited experimental validation scope.

The current experimental validation focuses mainly on the final warpage of printed specimens. While the comparison supports the model’s ability to reproduce topology-dependent deformation trends, in situ thermal measurements and full-field deformation monitoring were not included. Therefore, the present validation is more representative of trend-level agreement than of complete transient-field validation.

- Neglected process-related physical phenomena.

Some physical processes are not explicitly modeled, including melt flow, viscoelastic behavior, porosity formation, and possible variability in interlayer adhesion quality. These factors may further affect residual-stress accumulation and local deformation, particularly in regions with repeated reheating or strong geometric constraints.

- Applicability of the present framework.

The proposed method should be understood primarily as a toolpath-aware comparative thermo-mechanical modeling framework for evaluating the influence of infill topology and boundary configuration on warpage and residual stress in FDM. Further improvements in constitutive modeling, bead-shape representation, and experimental characterization are needed for a more quantitatively complete process simulation.

## 6. Conclusions

In this study, a G-code-driven, filament-level modeling and thermo-mechanical coupling framework was developed for the FDM process of complex lightweight structures. By directly parsing the slicing G-code, the deposited filaments were reconstructed through a path-aligned local coordinate system, and the progressive material addition was simulated using an element birth–death strategy based on centroid selection. On this basis, indirect thermo-mechanical coupling analysis was performed to predict the evolution of temperature, warpage, and residual stress during printing. According to the numerical and experimental results, the following conclusions can be drawn:

(1) The proposed G-code-driven framework improves the geometric fidelity and applicability of filament-level FDM simulation for complex toolpaths.

Compared with modeling methods restricted to paths aligned with the global coordinate system, the present local-coordinate-based reconstruction strategy can represent arbitrarily oriented in-plane deposition paths more robustly. In addition, the centroid-based element activation method reduces the dependence on strictly aligned hexahedral partitions and improves numerical adaptability for filament intersections and complex lightweight geometries.

(2) The thermal history is strongly governed by deposition sequence, path adjacency, and structural continuity.

For all investigated configurations, the high-temperature zone consistently follows the newly deposited paths, while previously printed regions cool progressively through conduction and convection. However, local reheating becomes more pronounced in regions with short turning segments, closely spaced adjacent paths, and filament intersections. After sidewalls and a bottom base are introduced, the continuous solid boundary provides an additional heat-conduction pathway and increases the local thermal mass, which elevates the thermal baseline and modifies the cooling behavior.

(3) Warpage is a cumulative consequence of repeated thermal cycling and non-uniform shrinkage, and its mode is highly dependent on infill topology and boundary configuration.

Both the triangular and open hexagonal infills exhibit deformation concentration near free outer boundaries and filament-intersection regions, indicating that open lattice structures release thermally induced shrinkage mainly through boundary freedom and local geometric discontinuities. In contrast, after the sidewalls and bottom base are introduced, the maximum displacement becomes the largest among the three cases, but the deformation is redistributed and constrained along the outer frame and internal support regions. This indicates that geometric closure does not necessarily reduce warpage magnitude, but rather changes the way in which shrinkage incompatibility is accommodated.

(4) Residual-stress evolution shows a clear trade-off between structural continuity and stress relaxation capability.

The triangular and open hexagonal infills exhibit relatively discrete stress concentrations and comparable stress levels, suggesting that open lattice topologies can partially release thermally induced deformation through geometric compliance. By contrast, the configuration with sidewalls and a bottom base develops much higher equivalent stress, mainly concentrated near the contact regions between the infill and the bottom-supported continuous solid. Therefore, although enclosed boundary structures improve contour integrity and structural continuity, they also increase cooling restraint and residual-stress accumulation.

(5) The experimental warpage measurements support the topology-dependent predictive capability of the proposed model.

The printed specimens reproduce the same main warpage tendencies as the simulations for all three configurations, and the relative differences between simulated maximum displacement and measured warpage remain within an acceptable range. This indicates that the present filament-level framework can capture the main deformation characteristics associated with different infill and boundary layouts, although some deviations in absolute values still remain due to the simplified constitutive, geometric, and thermal assumptions.

Overall, the present study shows that the thermo-mechanical response of FDM lightweight structures cannot be evaluated solely from geometric topology or printability considerations. Instead, the coupled effects of toolpath connectivity, local reheating, boundary continuity, and shrinkage constraint must be considered simultaneously. The proposed framework therefore provides a useful toolpath-aware simulation approach for comparative analysis and design of FDM lightweight structures.

Future work will focus on incorporating more realistic constitutive descriptions, including anisotropy and viscoelasticity, together with refined bead-shape representation, richer thermal boundary characterization, and more comprehensive in situ experimental validation, so as to further improve the quantitative predictive capability of the model.

## Figures and Tables

**Table 1:** Hardware and Software.

Category	Configurations
Slicing software	Ultimaker Cura 5.2.1
Simulation software	Mechanical APDL Product Launcher 2022 R1
Programming environment	CodeBlocks (language: C)
Printing material	Acrylonitrile–butadiene–styrene (ABS)
FDM 3D printer	BambuLab X1-Carbon model 3D printer

**Table 2:** Material parameters.

Temperature (°C)	Density (Kg/m^3^)	Thermal Conductivity W·(m·°C)^−1^	Specific Heat J·(kg·°C)^−1^	Elastic Modulus Pa	Coefficient of Thermal Expansion °C^−1^	Poisson’s Ratio
25	1050	0.225	1486	2.6 × 10^9^	8.5 × 10^−5^	0.394
50	1050	0.225	1684	2.5 × 10^9^	8.5 × 10^−5^	0.394
100	1050	0.225	2020	1.0 × 10^9^	8.5 × 10^−5^	0.394
150	1050	0.225	2080	0.6 × 10^9^	8.5 × 10^−5^	0.394
200	1050	0.225	2214	0.3 × 10^9^	8.5 × 10^−5^	0.394
250	1050	0.225	2330	0.3 × 10^9^	8.5 × 10^−5^	0.394

**Table 3:** Major process parameters for FEM.

Process Parameter	Values
Line width	0.4 mm
Layer thickness	0.2 mm
Number of layers	5
Nozzle temperature	220 °C
Printing speed	40 mm/s
Build plate temperature	35 °C
Chamber temperature	30 °C
Cooling conditions	Forced convection cooling

**Table 4:** Mesh sensitivity results for hexagonal infill.

Mesh Size (mm)	Elements Through Thickness	Max Displacement (mm)	Max Von Mises (Pa)
0.2	1	4.3812 × 10^−2^	1.74 × 10^7^
0.1	2	3.9194 × 10^−2^	1.89 × 10^7^

**Table 5:** Quantitative comparison of simulation and experiment for different infill configurations.

Configuration	Max Displacement (mm)	Experimental Warpage (mm)	Relative Difference	Max SEQV (Pa)
Square Plate with Triangular Infill	4.2696 × 10^−2^	5 × 10^−2^	14.6%	1.87 × 10^7^
Square Plate with Hexagonal Infill	4.3812 × 10^−2^	4 × 10^−2^	9.5%	1.74 × 10^7^
Square Plate with Hexagonal Infill, Sidewalls and Bottom Base	4.792 × 10^−2^	5 × 10^−2^	4.2%	3.82 × 10^7^

## Data Availability

The original contributions presented in this study are included in the article. Further inquiries can be directed to the corresponding author.

## Abbreviations

The following abbreviations are used in this manuscript:

FDM	Fused Deposition Modeling
CAD	Computer-aided Design
APDL	ANSYS Parametric Design Language
AM	Additive Manufacturing
ABS	Acrylonitrile Butadiene Styrene
DOE	Design of Experiments
FEA	Finite Element Analysis
FE	Finite Element
PLA	Polymerized Lactic Acid
SMN	Solution Min
SMX	Solution Max
SEQV	Equivalent stress
